# The international Hip Outcome Tool-33 (iHOT-33): multicenter validation and translation to Spanish

**DOI:** 10.1186/s12955-015-0255-z

**Published:** 2015-05-20

**Authors:** Miguel Angel Ruiz-Ibán, Roberto Seijas, Andrea Sallent, Oscar Ares, Oliver Marín-Peña, Alfonso Muriel, Ricardo Cuéllar

**Affiliations:** Unidad de Hombro y Codo, Hospital Universitario Ramón y Cajal, Cta Colmenar Km 9.100, Madrid, 28046 Spain; Artroscopia GC - Fundación García Cugat - Hospital Quirón Barcelona, Barcelona, Spain; Anatomy Department, Universitat Internacional de Catalunya, Sant Cugat, Spain; Hospital Vall d’Hebron, Barcelona, Spain; Hospital Universitario Infanta Leonor, Madrid, Spain; Unidad de Bioestadística Clínica, IRYCIS Instituto Ramón y Cajal de Investigación Sanitaria, Madrid, Spain; CIBERESP Centro de Investigación Biomédica en Red de Epidemiología y Salud Pública, Madrid, Spain; Hospital Universitario Donostia, San Sebastián, Spain

## Abstract

**Background:**

The international Hip Outcome Tool-33 (iHOT-33) is a 33-item self administered outcome measure based on a Visual Analogue Scale response format designed for young and active population with hip pathology. The aim of the present study is to translate and validate the iHOT-33 into Spanish.

**Methods:**

97 patients undergoing hip arthroscopy were included in this prospective and multicenter study performed between January 2012 and May 2014. Crosscultural adaptation was used to translate iHOT-33 into Spanish. Patients completed the questionnaire before and after surgery. Feasibility, reliability, internal consistency, construct validity (correlation with Western Ontario and McMaster Universities Osteoarthritis Index), ceiling and floor effects and sensitivity to change were assessed for the present study.

**Results:**

Mean age was 48 years old. *Feasibility*: 41.2 % patients had no blank questions, and 71.3 % of patients had fulfilled all but one or two questions. *Reliability*: ICC for the global questionnaire was 0.97, showing that the questionnaire is highly reproducible. *Internal consistency*: Cronbach’s alpha was 0.98 for the global questionnaire. *Construct validity*: there was a high correlation with WOMAC (correlation coefficient >0.5). The *Ceiling effect* (taking into account the minimum detectable change) was 12.1 % and the floor effect was 21.6 %, for the global questionnaire. Large *sensitivity to change* was shown.

**Conclusion:**

the Spanish version of iHOT-33 has shown to be feasible, reliable and sensible to changes for patients undergoing hip arthroscopy. This validated translation of iHOT-33 allows for comparisons between studies involving either Spanish- or English-speaking patients.

**Level of evidence:**

Prognostic study, Level I.

## Introduction

The prevalence of hip injuries in the young adult population is significant. In the last decades there has been significant advances in the identification and treatment of different pathologic conditions that affect the young adult hip such as labral tears, cartilage injury, capsular/iliofemoral ligament injury, femoroacetabular impingement, hip instability and athletic pubalgia [1–3].

Questionnaires are a key tool in orthopedic surgery, as well as in other many specialties, in order to assess the impact that any procedure has on patients’ daily life and correctly evaluate what impact any treatment protocol has in a specific pathology. The classic tools designed to evaluate results in patients with hip pathology (sucha as the Harris Hip Score [4] or the Western Ontario and McMaster Universities Osteoarthritis Index [5] (WOMAC) score) were initially designed to evaluate patients with hip osteoarthritis (OA) and had significant functional impairment, these tools have limtations when used to assess problems in younger adults with subtle hip dysfunction that are however functionally significant for them [6].

A recent meta-analysis by Thorborg et al. suggest that a new patient-reported outcome questionnaire should be developed in younger patients with hip and/or groin patients [7], in line with the study by Tijssen et al. [8]. Other authors have stated the benefits of hip arthroscopy for intra-articular pathology, although more specific tools should be used to study the impact of surgery [9]. To date, the International Hip Outcome Tool-33 (iHOT-33) questionnare is the first addressed to young and active patients with hip disorders. The iHOT-33 is a 33-item self administered outcome measure based on a VAS response format designed for young and active population with hip pathology [6]. iHOT-33 has shown to be reliable and shows face, content, and construct validity, as well as being highly responsive to clinical change [6]. Harris-Hayes et al. stated iHOT-33, together with Copenhagen Hip and Groin Outcome Score scored the best when assessing treatment of FAI [10].

The vast majority of questionnaires for hip problems have been developed in English, and therefore must undergo a validated translation that is mandatory for its use in a language different from the one in which it was developed [11–14]. To our knowledge, iHOT-33 has only previously been translated to Portuguese, although the authors did not perform a validation [15]. The aim of this study is to translate the iHOT-33 questionnaire into Spanish and to validate the translation in order to provide health care professionals in all Spanish-speaking countries with a more specific evaluation tool for young patients with hip disorders.

## Methods

The iHOT-33 questionnaire is divided into four sections; 1) Symptoms and functional limitations, 2) Sports and recreational activities, 3) Job related concerns, and 4) Social, emotional and lifestyle concerns [6]. Questions are evaluated according to the 100-point Visual Analogue Scale format previously used in other questionnaires [16, 17]. This score does not require mathematical transformation and is easily understood. Each question scores out of 100, 0 representing the worst possible quality-of-life score and 100 being the best score possible. Summing up the scores from all questions and dividing them by the number of questions answered determines the patient’s final score out of 100, it is also easy to calculate an independent score for each domain [16, 17].

### Crosscultural adaptation

There is a well-established protocol in order address the translation of health-related questionnaires within different languages; the crosscultural adaptation of a questionnaire tries to assure a perfect equivalence from the original form [18–21]. This process refers to the translation as well as to the transcultural adaptation, thus adapting the evaluation outcomes according to different cultures and is summarized as follows:

1. Forward translation of the original iHOT-33 (English) into Spanish, by two independent professional translators (one English-native and one Spanish-native).
2. Review of the translations and synthesis of the first draft (version 0.1)
3. Back-translation of version 0.1 in Spanish to English by two English-native translators.
4. Review of both the back and forward translations. Drafting of the second version in Spanish (version 0.2) by an expert linguistic translator specialized in medical questionnaires and by a third translator.
5. Pretesting of the work (version 0.2) by a panel of 4 orthopedic physicians and 30 patients to assure that the text could be understood. Writing of version 1.0 (final version, see Appendix 1).

Patients included in the present study completed version 1.0 of the questionnaire and all statistical analysis of the psychometric parameters was performed upon this version 1.0.

### Patients

A prospective study with 100 patients was performed between January 2012 and May 2014 in order to carry out the transcultural adaptation and a validation of the iHOT-33.

Four surgeons from different medical centres were involved in recruiting 25 patients each. Patients were included if they were between the ages of 18 and 60, and had symptomatic hip pathology for at least 6 months which required surgical treatment and had it scheduled. Patients were informed that their data from questionnaires would be used for this research and written and oral consent was obtained. Although 100 patients were initially recruited for the present study, three patients were lost during follow-up and thus 97 were the final number of patients included. The patients were consecutively recruited in each surgeon clinic and included in the study when they fulfilled the inclusion criteria, signed the written consent form and undertook the surgical procedure.

The patients were given a questionnaire that included a copy of the translated iHOT-33 scale and a copy of the Spanish version of the WOMAC [13] and were asked to fulfill it in clinic. They were also provided with a second blank copy of the questionnaire with an stamped and addressed envelope with instructions to fulfill it again in 15 days and send it back to the investigators. A third copy of the questionnaire was fulfilled by the patients who been operated of their hip problems and were evaluated 6 months after the initial assessment. The WOMAC was used to test construct validity as it has been previously translated and validated in Spanish [13, 22]. This questionnaire evaluates pain, stiffness and function with five difficulty-based response options in patients with hip and/or knee OA [7]. A lower score on the WOMAC indicated a better quality of life (vice versa in the iHOT questionnaire). Once the three subscales are added up, data was standardized to a range from 0 to 100 (being 0 the best health status and 100 the worst).

### Statistical analysis

Feasibility, reliability, internal consistency, construct validity (correlation with WOMAC), ceiling and floor effects and sensitivity to change were assesses for the present study, in concordance with previous validation-related articles [6, 12, 14]. All statistical analysis was performed with SPSS statistical software version 21.0 (Chicago, IL, USA).

#### Feasibility

This parameter refers to the proportion of patients that did not answer any item, according to the preoperative visit. Feasibility was analyzed in the 97 questionnaires fulfilled in the first visit. The expected missing items proportions were similar to those obtained by previous validated translations of other questionnaires, as no feasibility was calculated for the original iHOT-33 questionnaire [14, 23, 24].

#### Reliability

A 15-day test-retest reliability was applied to the present manuscript. Of the 97 patients that fulfilled the initial translated version of iHOT-33. 73 sent back copies fulfilled 15 days after the initial evaluation.

Test-retest reliability was determined using intraclass correlation coefficient (ICC) (two-way random effects model) [25] as well as standard error of measurement (SEM) and represented using a Bland-Altman plot. According to the previously published by Mohtadi et al. [6], ICC scores were expected to be >0.78. In order to assess results, the minimal value considered acceptable for ICC was 0.78. Minimal detectable change (MDC) responded to the following formula: MDC = SEM × 1.4142 × 1.9 [26]. This expresses the degree of change required in an individual’s score in order to consider it as ‘real’ and not due to measurement errors.

#### Internal consistency

Cronbach’s α is used to measure internal consistency and a questionnaire is usually considered as consistent when α >0.8 [27]. Internal consistency was analyzed in the 97 questionnaires fulfilled in the first visit.

#### Construct validity

Defined as the degree to which an instrument measures the characteristic being investigated. This was measured comparing the results obtained in the 97 questionnaires fulfilled in the first visit in both scales iHOT-33 and WOMAC [13, 22]. Construct validity was assessed with a correlation analysis between both scales using the Spearman’s Rho. A threshold of r > 0.5 is considered acceptable suggesting moderate to high correlation [27]. WOMAC values were first reversed as these two scales are orientated in opposite directions in order to obtain positive values.

#### Ceiling and floor effects

The ceiling effect refers to the percentage of patients with maximum score within the questionnaire, indicating the best clinical outcome. On the other hand, the floor effect accounts for the proportion of patients with a minimum score, showing the worst clinical outcome. Ceiling and floor effects can be worked out as percentage of patients with maximum or minimum scores, respectively, or either with the maximum score (97 points in this case) minus the minimal detectable change (MDC) and worst score (0 points) plus the MDC, respectively. Within the present manuscript, both methods were used to describe these effects.

#### Sensitivity to change

All 97 patients were available for evaluation with the questionnaire after surgery and 6 months after the initial evaluation. The differences in mean scores before and after surgery at 6 months postoperative, using paired *t*-test or Wilcoxon signed-ranked test using an analysis for homogeneous samples with homogeneous expected change [28]. The ability of an instrument to detect change is quantified dividing the mean change by the standard deviation in change: the standardized response mean (SRM) [29]. SRM values of 0.20, 0.50 and 0.80 represent small, moderate and large sensitivity to change, respectively [30]. Effect size (Cohen’s d) was also assessed to evaluate the extent of change and to allow comparison between questionnaires; it is calculated as the difference between the mean preoperative and postoperative scores, divided by the standard deviation of the preoperative scores. An effect size of 1.0 equals a change of one standard deviation in the sample [31]. As there is no external standard against which to measure functional capacity we employed an analysis for homogeneous samples with homogeneous expected change. The statistical coefficients used were based on group-level effect sizes, including the mean response (SRM: mean change/standard deviation for change).

## Results

A total of 37 women and 60 men with a mean age was 43.8 years old (SD 10.9, range 22 to 60 years) were included in the study (three patients lost during follow-up from the initial 100 included). The patients had the following clinical diagnosis: FAI (78; 65 combined impingement, 11 Cam-type lesions, 2 Pincer-type lesions), OA (10), gluteous medius pathology (3), Perthes sequelae (2), slipped capital femoral epiphysiolysis sequelae (2), psoas tendinitis (1), developmental dysplasia of the hip (1), and osteonecrosis (1).

### Cross-cultural adaptation

No major problems were observed during forward and back-translation of iHOT-33 with language or grammatical errors. Small discrepancies rose for many synonyms but were easily agreed during revision. Pre-testing of version 0.2 revealed no further complications or comprehension issues and was thus upgraded to version 1.0.

#### Feasibility

Ninety-seven questionnaires were studied for feasibility (Table 1). 40 patients (41.2 %) filled out the entire questionnaire. 30 patients left either one or two questions without answering, thus, 71.3 % of questionnaires collected had a maximum of two blank questions.

**Table 1:** Feasibility of the International Hip Outcome Tool-33 (iHOT-33); number of missing items registered within our questionnaires

	0 missing items	1 missing item	2 missing items	3 or more missing items
**Symptoms and functional limitations** (questions 1–16)	94	3	0	0
**Sports and recreational activities** (qq. 17–22)	72	24	0	1
**Job related concerns** (qq 23–26)	59	24	1	13
**Social, emotional and lifestyle concerns** (qq 27–33)	60	25	12	0

#### Reliability

**Fig. 1:**
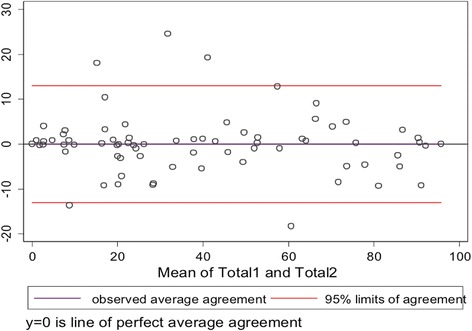
Bland-Altman plot for the studied scores of the iHOT-33

All subscales obtained excellent ICC within the 15-day test-retest reliability; 0.95 (CI 95 %; 0.92 to 0.98) for the Functional subscale, 0.92 (CI 95 %; 0.76 to 0.98) for the Sports subscale, 0.93 (CI 95 %; 0.83 to 0.98) within the Job subscale and 0.96 (CI 95 %; 0.91 to 0.98) for the Social subscale. ICC for the global questionnaire was 0.97 (CI 95 %; 0.96 to 0.99). Mean scores for all subscales and globally at the test and retest are shown in Table 2. The SEM was ±4.66 for the iHOT-33 questionnaire. Thus, MDC was 12.5 points (Fig. 1).

**Table 2:** Mean scores at the 15-day test-retest questionnaires

	Mean score test	Mean score 15-day retest
**Functional**	43.12	43.66
**Sports**	28.81	28.37
**Job**	37.15	36.57
**Social, lifestyle**	40.64	40.06
**TOTAL**	39.37	40.09

#### Internal consistency

Cronbach’s α for the global questionnaire was 0.98, confirming a high internal consistency. Furthermore, Cronbach’s α was scored for the different subscales: 0.97 for Functional subscale, 0.94 Sports subscale, 0.89 Job subscale, and 0.94 Social and lifestyle subscale.

#### Construct validity

iHOT subscales showed a moderate to high construct validity against the WOMAC score (Table 3) in all subscales, being statistically significant. iHOT –functional subscale showed the highest validity with WOMAC.

**Table 3:** Construct validity showing the correlations between International Hip Outcome Tool-33 (iHOT-33) and WOMAC questionnaires

		iHOT-33			
		Functional	Sports	Job	Social
**WOMAC**	**Pain**	0.744	0.526	0.581	0.653
	**Stiffness**	0.687	0.513	0.544	0.574
	**Function**	0.79	0.536	0.616	0.658

All correlations were statistically significant (<0.001)

#### Ceiling and floor effects

Taking into account the MDC, ceiling effect was observed in 12.1 % of patients whereas floor effect accounted for 21.6 % of patients. When only the best (100 points) and worst (0 point) scores were considered, 2 patients were found with floor effect and 0 ceiling effect.

#### Sensitivity to change

SRM score was 1.18, showing large sensitivity to change. Cohen’s d (pooled variance) was 1.46.

## Discussion

The present study aimed to translate and validate the International Hip Outcome Tool-33 (iHOT-33) to Spanish. Given the abovementioned results, a correct cross-cultural adaptation and posterior validation has been proven, showing that the iHOT-33 questionnaire can be used in Spanish-speaking countries.

Health-related questionnaires are a means of quantifying a subjective experience, aiming to provide the professional with patients’ satisfaction and quality of life information following surgical or nonsurgical treatments. The WOMAC questionnaire was the only validated and hip-specific questionnaire in Spanish available for surgeons treating younger active patients with hip problems in Spanish speaking countries [13], whereas English-speaking countries enjoy of more validated questionnaires and scores. This study has allowed for the development of such a tool.

Recently, our group has developed the Spanish translation and validation of the Hip Outcome Score (HOS); a hip-specific questionnaire designed for evaluating outcomes following hip arthroscopy [14]. In contrast to the iHOT-33, the HOS questionnaire was designed to measure functional or sport physical limitations but did not include either emotional, social or lifestyle dimensions, nor dies it assess the impact of the patients’ problems on their jobs [6]. When compared to the Spanish validation of the HOS both had similar reliability, internal consistency, sensibility to change and construct validity [14] but the multidimensional nature of the iHOT-33 might make it more valuable in assessing these patients.

The questionnaire’s *feasibility* was generally good, however, only 41.2 % of patients fulfilled the entire questionnaire. When analysed separately, the subscale with more questions answered was the Symptoms and functional limitations; only 3 patients left one question blank. On the other hand, 24 patients had one unanswered question within the Job subscale and 13 had three or more. This can be explained in part by the high unemployment observed in Spain during the development of the present study, and by the reluctance of patients to express their more social and emotional concerns, whereas the functional outcomes subscale is a more direct-answer, pathology-related questions. In the present validation, the questions with more missing items were number 23 (How much trouble do you have pushing, pulling, lifting or carrying heavy objects at work?) and 32 (How concerned are you about picking up or carrying children because of your hip?). This is related to the number of patients that do not perform these activities. In further studies, the iHOT-12 would warrant these answers, as this shorter questionnaire requires the patients to answer all questions, imagining how would their hip feel even though they have not performed that activity [32].

The questionnaire showed an excellent reliability with ICC scores over 0.90 in all subscales including the overall score, in line with the original ICC scores published by Mohtadi et al., as for Cronbach’s α, showing high internal consistency [6]. Excellent correlation was obtained between the iHOT-33 and the WOMAC, especially within the Functional outcomes subscale. The original validation compared the iHOT-33 to the Non-Arthritic Hip Score (NAHS), observing very good correlation [6].

Regarding the ceiling and floor effects; only 0 and 2, respectively, were observed in the present study, whereas the original validation did not find any [6].

### Limitations

Several limitations should be taken into consideration with respect to this study. First, the present questionnaire has a shorter form (iHOT-12), which has shown to be reliable, valid and responsive to change [32]. The present translation only took the original iHOT-33 for validation, despite the short form could also be useful for preoperative visit and follow-ups. However, the decision was made to translate the original in order to start from the very beginning. Further studies could be addressed to this short form.

Secondly, despite the present study was a multicenter, all hospitals involved were located in Spain. Thus, some words of the translated version should be reviewed when administering the questionnaire in other Spanish-speaking countries. Despite having an official organization that regulates the Spanish language (Real Academia Española), local colloquialisms are extraordinarily frequent due to the extensive geographic distribution of the Spanish language and the high number of available words.

Furthermore, only four Spanish hospitals were included in the collection of data. However, the different clinical scenarios of the hospitals involved (combining private practice, cosmopolitan public hospitals and smaller regional hospital, as well as populations form both urban or rural areas) guarantees a well-distributed inclusion criteria in order to avoid socioeconomic or cultural bias.

Last, a greater number of patients could have been collected for the present study, however, the number of patients collected for this validation is clearly in line with other validation attempts in hip pathology previously published and the original validation of the iHOT-33 [14, 24].

In conclusion, this translated and validated Spanish version of the iHOT-33 has a valid construction; it has also high reliability, feasibility and has a large sensitivity to change with significant internal consistency in patients with hip disability. This validation of the iHOT-33 allows health care professionals to evaluate results between Spanish-speaking.
